# Application of next generation sequencing in oncology

**DOI:** 10.1186/1471-2164-15-S2-O7

**Published:** 2014-04-02

**Authors:** Brian Meyer

**Affiliations:** 1 King Faisal Specialist Hospital and Research Center, Riyadh, Saudi Arabia

## 

Oncology is an area of clinical practice at the forefront of personalized medicine which has been facilitated by advances in genomics. These were initially driven by microarray technologies and subsequently by Next-Generation Sequencing (NGS). In both instances advances were and are highly dependent upon parallel progress in High Performance Computing and Bioinformatics. Clinical Laboratories have already engaged NGS in simultaneously determining the mutational status of tumor in relation to multiple candidate genes. NGS is particularly suited to oncology testing because of its sensitivity. Often, as a consequence of tumor heterogeneity or the presence of normal cells in tumor biopsies, somatic mutations are present in only a fraction of cells analyzed. Screening of a limited number of genes in clinical practice frequently results in identifying mutations which predict response to targeted therapies (e.g. activating EGFR mutations in lung cancer). Similarly, many of the genes screened have prognostic value or in some instances are able to yield a more definitive diagnosis. However, at a research level the technology has progressed even more rapidly and has enabled a systematic cataloguing of cancer genomes. Through the efforts of international projects such as the Cancer Genome Atlas and the International Cancer Genome Consortium, recurrent mutations, translocations and therapeutic targets are regularly reported in relation to specific cancer subtypes. The translation of this torrent of data and the need for continuous adaptation to new technologies provide significant challenges. Despite these it remains clear that the molecular characterization of tumors through technologies such as NGS for diagnostic, prognostic and therapeutic purposes is very much a current part of clinical practice, will expand rapidly, and will be a driving force for the application of personalized medicine particularly in the field of oncology.

